# Cytotoxicity of gemcitabine-loaded thermosensitive liposomes in pancreatic cancer cell lines

**Published:** 2015-04-16

**Authors:** Kevin Affram, Ofonime Udofot, Edward Agyare

**Affiliations:** Division of Basic Pharmaceutical Sciences, College of Pharmacy and Pharmaceutical Sciences, Florida A&M University, USA

**Keywords:** liposomes, gemcitabine, hyperthermia, nanoparticles, pancreatic cancer

## Abstract

Gemcitabine (GEM) is currently the standard option for the treatment of pancreatic cancer but its short half-life and rapid metabolism has caused for new modality for delivery of GEM. The purpose of this study was to formulate GEM loaded PEGylated thermosensitive liposomal nanoparticles (GEM-TSLnps) to increase residence time and deliver high payload of GEM to pancreatic cancer cells using mild hyperthermia (mHT). The GEM-TSLnps were formulated by thin film hydration. The cytotoxic effects of GEM and GEM-TSLnps were evaluated against human pancreatic cancer cell lines. In vitro release of GEM by TSLnps was determined at temperatures from 26°C through to 50°C. Cell viability studies, clonogenic assay, flow cytometry and confocal imaging were performed on pancreatic cancer cell lines using GEM and GEM-TSLnps + mHT. The GEM-TSLnp size was determined to be 216.10 ± 0.57 nm with entrapment efficiency of 41.10 ± 2.0%. GEM release from TSLnps was sharply increased at 42°C (60%) than at 37°C (25%), (p<0.01). *In vitro* cytotoxicity of GEM-TSLnps + mHT treated pancreatic cancer cell lines was significantly higher than GEM treated. The IC50 values for PANC-1, MiaPaCa-2 and BxPC-3 cells GEM-TSLnps + mHT treated were 1.2 to 3.5 fold-higher than GEM treated. Among the cell lines, GEM-TSLnps + mHT treated PANC-1 and MiaPaCa-2 cells show significantly reduced reproductive viability compared with the GEM treated cells. Flow cytometric and confocal images revealed high Rho-TSLnps cellular uptake. Our findings suggest that GEMTSLnps+ mHT can significantly enhance cytotoxic effect of GEM and could serve as a new chemotherapy modality for delivering GEM.

## Introduction

Pancreatic cancer is one of the most aggressive human malignancies, with a yearly incidence that equals its mortality. An estimated 48,960 new cases of pancreatic cancer are expected to occur in the US of which 40,560 deaths are expected in 2015, about the same number in women (19,850) as in men (20,710) [1,2]. The survival rate remains stagnant between 5–6% in the first 5 years with high mortality rate within the first year [2]. There are no screening tests for pancreatic cancer and early diagnosis is difficult because pancreatic cancer often develops without any symptoms [3]. Rapid growth, early metastatic dissemination to distant sites, and resistance to most tumor-directed and systemic therapies, makes the management of pancreatic cancer highly challenging [4]. Given that less than 10% of all pancreatic cancer patients can truly undergo curative resection and the high incidence of life threaten complications such as damage to liver is imminent, innovative delivery of chemotherapy capable of surmounting biological barriers and release their payloads deep in the tumors becomes important option [5,6]. Localized therapies are therefore a critical component of treatment – hence the renewed interest in an effective chemotherapy delivery.

A widely employed clinical strategy to improve therapy outcomes is to utilize newer chemotherapy drugs approved for the treatment of metastatic disease. For pancreatic cancer, the most promising drug is gemcitabine (GEM), a pyrimidine analog of deoxycytidine, which was shown to have modestly better response rates (clinical and radiographic) and survival rates than 5-fluorouracil, the previous standard-of-care [7]. However, despite potent preclinical activity by itself, it has not shown comparable clinical activity as a chemotherapeutic agent. The putative reason for this is GEM’s short plasma half-life (<20 min) due to conversion to its inactive metabolite dFdU by plasma deaminases deoxynucleotide [8], hence larger doses than normally required is given clinically to achieve therapeutic efficacy rendering patients to GEM induced systemic toxicity such as thrombolytic microangiopathy [9,10]. This rapid plasma degradation can be tackled by encasing GEM in a liposome, which is FDA approved drug carrier with high drug payload, and rendering it some stealth properties to evade capture by the reticuloendothelial system.

There still remains the issue of transporting this liposome past physical and biological barriers within pancreatic cancer [11]. Immature and leaky tumor vasculature typically leads to heterogeneous vascular perfusion within tumors - the invasive periphery of the tumor having the highest microvascular density and the tumor core being under perfused and thus, underexposed to drugs. The dense stromal component (desmoplasia) of pancreatic cancer accentuates hindrance of nutrient, oxygen and drug delivery by the physiological barriers already imposed by the interstitial matrix cancer [11–14]. The consequent hostile microenvironment of the tumor core harbors the most aggressive tumor cells with the greatest potential to regenerate if they survive cytotoxic treatment [6]. Amplifying this inherent aggressiveness is the acquired treatment resistance conferred by insufficient drug exposure [15].

Mild hyperthermia (mHT) heating of tumor tissue to temperatures of up to 42°C in conjunction with liposome results in effective drug accumulation and significant tumor growth suppression. This can be attributed to synergistic effect compare to the use of chemotherapeutic agent alone [16]. mHT over the years has yielded momentous improvement in therapeutic response among patients when used in combination with chemotherapeutic agents. mHT can be used to improve chemotherapy in two ways: i) to increase vascular permeability in solid tumors prior to intravenous administration and may therefore increase levels of liposome accumulation, and ii) to trigger release of drug from liposome[16]. By applying these two strategies, drug delivery to tumors can be strongly enhanced.

In the study we report cytotoxicity of GEM-TSLnps on pancreatic cancer cell lines (MiaPaCa-2, BxPC-3, AsPC and PANC-1) with mHT *in-vitro*. We evaluated the cytotoxicity of TSLnps with highest GEM entrapment based on cell viability, cell survival and IC_50_ with exposure to heat at 42°C for 10 min compared with free GEM. Overall, our study provides avenue towards using GEM-TSLnps and mHT to increase anticancer activity of GEM.

## Materials and methods

### Materials

Dipalmitoylphosphatidilcholine (DPPC),1-myristoyl-2-palmitoyl-sn-glycero-3-phosphocholine (MPPC), 1,2-distearoyl-sn-glycero-3-phosphoethanolamine-N-[amino(polyethylene glycol) 2000 (DSPE PEG_2000_), 1,2-distearoyl-sn-glycero-3-phosphacholine (DSPC), Cholesterol (CHOL) and 1,2-dipalmitoyl-sn-glycero-3-phosphoethanolamine-N-(lissaminerhodamine B sulfonyl) (ammonium salt) (DOPERho) were purchased from Avanti Polar Lipids (Alabaster, AL). Gemcitabine hydrochloride (2′-Deoxy-2′,2′-difluorocytidine) was bought from Sigma-Aldrich (St. Louis, MO). MiaPaCa-2, BxPC-3, AsPC and PANC-1 cell lines were purchased from American Type Culture Collection (ATCC) (Manassas, VA). All solvents used were of analytical grade.

### Liposome preparation

GEM loaded thermosensitive liposomal nanoparticles (GEMTSLnps) made of different molar ratio of lipids was prepared using thin film hydration method and the results summarized in Table 1. For each batch, 50 mg of lipid combination was dissolved in chloroform, and stream of dry nitrogen gas was passed through the solution to evaporate the chloroform. Residual chloroform was removed by drying the lipids under vacuum for 24 hr. The lipid thin film formed was hydrated with 2 ml of phosphate buffered saline (PBS, 1x) containing10 mM GEM-HCl, followed by vortexing intermittently for 15 min. The multi laminar vesicles formed were extruded through a 200 nm polycarbonate membrane (Whatman^®^Nuclepore Track-Etch Membrane) sandwiched between two presoaked filter papers for 15 times extrusions to form uniformed unilaminar vesicles/liposomes. The GEM entrapped liposome suspensions were dialyzed against PBS overnight using a dialysis membrane (Spectra/Por^®^ Dialysis Membrane MWCO 3500, Spectrum, Rancho Dominguez, CA) to remove any free GEM [17–19]. Similarly, Rho-TSLnps was prepared with DPPC, MPPC, DSPG_2000_, DOPE-Rho in a molar ratio of 90:10:3.5:0.5 using the above method. The final products, GEM-TSLnps and Rho-TSLnps, were lyophilized, kept in sealed cryovials and stored at 4°C in wrapped aluminium foil until use.

### Characterization of liposomes

Mean hydrodynamic particle sizes and zeta potentials of the various liposomal formulations were determined by dynamic laser light scattering (Particle Sizing systems, Santa Barbara, California). Each formulation was prepared in triplicate and the particle size and zeta potential measurement for each formulation was repeated three times (3x).

### Entrapment efficiency

To quantify the amount of GEM entrapment in each formulation, 1 ml of liposome suspension containing 20 mg of GEM-TSLnp was diluted with 1ml of 30% triton-X and made up to 3 ml with mobile phase (mobile phase 10 mM phosphate buffer, pH 3.0 containing 5% of acetonitrile). The resulting solution was vortexed for 1min and centrifuged at 10,000 rpm (9,500 rcf) for 10 min. The amount of GEM present in supernatant was determined using HPLC (column=C18, 4.6×250 mm, flow rate of 1.0ml/min, injection volume 20 μl). The entrapment efficiency (EE) was calculated as shown below [20]

$$\%\;\text{EE}=\frac{\text{Amount}\;\text{of}\;\text{GEM}\;\text{entrapped}}{\text{Initial}\;\text{amount}\;\text{of}\;\text{GEM}\;\text{added}}\times 100$$

### *In vitro* release of GEM-TSLnps

The release of GEM from TSLnps at various temperatures (23, 34, 37, 39, 42, 45, and 50°C) was determined. Briefly, 20 mg of lyophilized GEM-TSLnps was suspended in 1 ml of PBS in a dialysis bag (MWCO:12,000 Daltons) and placed in a screw cap top glass bottle containing 5 ml PBS. It was then heated to a set temperature with gentle and continuous stirring (80 rpm) and maintained for 10 min. After 10 min, amount of GEM released into the receiver medium (PBS) was analyzed using HPLC as described above. Total amount of GEM entrapped in TSLnps was determined by disrupting them with 30% triton-X and diluted to suitable volume with mobile phase. The percentage release at each temperature was calculated relative to total amount of GEM in disrupted liposomes [19].

### Cell viability studies

The trypan blue assay was used to determine the cytotoxicity of free GEM and GEM entrapped liposomes (GEM-TSLnps). MiaPaCa-2 and PANC-1 cell lines were cultured using DMEM while BxPC-3 and AsPC cells were grown using RPMI 1640 medium. All the media were supplemented by L-glutamine, 1,000 mg/L glucose and 10 mM HEPES buffer supplemented with 10% fetal bovine serum (FBS) and 5 ml penicillin-streptomycin (10,000 U/ml). Exponentially growing of each cell line was plated out at a seeding density of 2.0×10^4^ cells per well in 12-well plates in triplicates and incubated at 37°C, (pCO_2_, 5%; humidity, 95%). All cell lines were allowed to reach 70% confluence prior to treatment. Cells were treated with varying amount of GEM and GEM-TSLnps and incubated for 24 hr. After 24 hr, GEM-TSLnps treated cells heated by placing the 12-well plates in precision-controlled temperature digital incubator at 42°C (± 0.02°C) for 10 min (after the incubator has reached thermal equilibrium), then returned back to 37°C (pCO_2_, 5%; humidity, 95%). After 48 hr, cells were detached with 0.25% trypsin-EDTA solution, centrifuged for 3 min at 2,500 rpm. The pellet obtained was gently re-suspended in growth medium. An aliquot of suspended cells were then mixed with 0.4% trypan blue (1:1) and percent live cells was counted using the Bio-rad automated cell counter (Hercules, CA) [21].

### Clonogenic assay

To determine cell survival *in-vitro* after treatment, each cell line was plated at a seeding density of 1.5–2.0×10^5^ cells per 25 cm^3^ flask and incubated at 37°C (pCO_2_, 5%; humidity, 95%) until cells reached 70–75% confluent. Each flask was then treated with varying amount of GEM and GEM-TSLnps. After 24 hr, GEM-TSLnps treated cells were exposed o heat at 42°C for 10 min and then placed back in CO_2_ incubator at 37°C. On the 7^th^ day, cells were trypsinized with 0.25% trypsin-EDTA solution, centrifuged, and pellets re-suspended in culture medium. PANC-1, MiaPaCa-2 and AsPc cell lines were plated in triplicates in 6-well plates with 1,000 lives cells in each well, whereas BxPC-3 cells were plated with 2,500 live cells. The cell lines were incubated at 37°C (pCO_2_, 5%; humidity, 95%). After a period of days (BxPC-3 were incubated for 8 days, MiaPaCa-2 cells for 10 days, AsPC for 14 days, and PANC-1 cells were incubated for 16 days), formed colonies were fixed, stained and counted. Colonies with less than 50 cluster of cells were not counted [22].

### *In vitro* cellular uptake of GEM-TSLnps

#### Confocal imaging

MiaPaCa-2, BxPC-3, AsPC or PANC-1 cells were grown on cover slips at cell density of 1.5×10^5^ in duplicates in 6-well plates and stabilized for 24 hr at 37°C. Cells were then treated with 1 ml of 0.5% TSLnps rhodamine labeled liposome nanoparticles (Rho-TSLnps) in growth medium and incubated for 4 hr at 37°C. Prior to the end of incubation, cells were washed twice with PBS (pH 7.4) and DAPI was (0.75 μg/ml) was added for nuclear staining [23]. After incubation, cells were washed twice with PBS and fixed with 500 μl of 4% paraformaldehyde for 15min at room temperature and washed twice with PBS [24]. The intracellular localization of Rho-TSLnps was observed by confocal laser microscope.

#### Flow cytometric analysis

Cells (MiaPaCa-2, BxPC-3, AsPC and PANC-1) were seeded in 6-well plates in duplicates at 2.0×10^5^ cells per well in growth medium and incubated for 48 hr before treatment with 1 ml of 0.5% Rho-TSLnps for 2 hr at 37°C. At the end of Rho-TSLnps exposure, medium was removed and the cells washed twice with PBS and harvested. The cell suspension was centrifuged for 3 min at 2,500 rpm and the pellets were fixed with 4% paraformaldehyde (PFA). Cells were then washed three times with PBS and finally suspended in 500 μl PBS[24], and immediately analyzed with FACS caliber cytometer using 488 nm laser for excitation of Rho and a band centered at 585 nm for detection a fluorescence.

### Statistical analysis

The difference between GEM and GEM-TSLnps treated groups were analyzed using Student’s t-test (paired) and considered significant at p<0.05. All experiments were performed at least in triplicate and analyzed using GraphPad Prism software (GraphPad Software, Inc., La Jolla, CA, USA).

## Results

### Preparation and characterization of liposomes

Five (5) batches of different thermosensitve liposomes were prepared (Table 1). Among all the batches; GEM-TSLnp has the highest entrapment efficiency of GEM of 41.10%, while GEM-TSLnp_3_ and GEM-TSLnp_4_ were determined to have the lowest comparable GEM entrapment efficiency of 3.01% and 2.94% respectively. Also, GEM-TSLnp_1_ and GEM-TSLnp_2_ entrapment efficiency of GEM were found to be 9.48% and 7.73% respectively with a difference of 1.75%.

For the mean particle size, GEM-TSLnp_2_ has the largest mean particle size (285 ± 0.442 nm) followed by GEM-TSLnp (216.10 ± 0.565 nm) and GEM-TSLnp_1_ (204.80 ± 0.499 nm) even though GEM entrapment efficiency for GEM-TSLnp_2_ was far lower than GEMTSLnp (Table 1). As expected, GEM-TSLnp_4_ has the lowest particle size (155.80 ± 0.320 nm) and closely followed by GEM-TSLnp_3_ (159.70 ± 0.388 nm). The low particle sizes may likely due to their low GEM entrapment efficiency. Although GEM-TSLnp_4_ exhibited lowest particle size and GEM entrapment efficiency, its zeta potential value was the highest (1.090 ± 0.023 mV) whereas a negative zeta potential (−0.047 ± 0.117 mV) was observed for GEM-TSLnp. The zeta potential values for GEM-TSLnp_1_, GEM-TSLnp_3_ and GEM-TSLnp_2_ batches were shown as 0.623 ± 0.110 mV, 0.153 ± 0.025 mV and 0.018 ± 0.678 mV respectively. Based on the entrapped efficiency, GEM-TSLnp batch was selected as the final product for all the studies.

### *In vitro* release behavior of GEM-TSLnps

**Figure 1:**
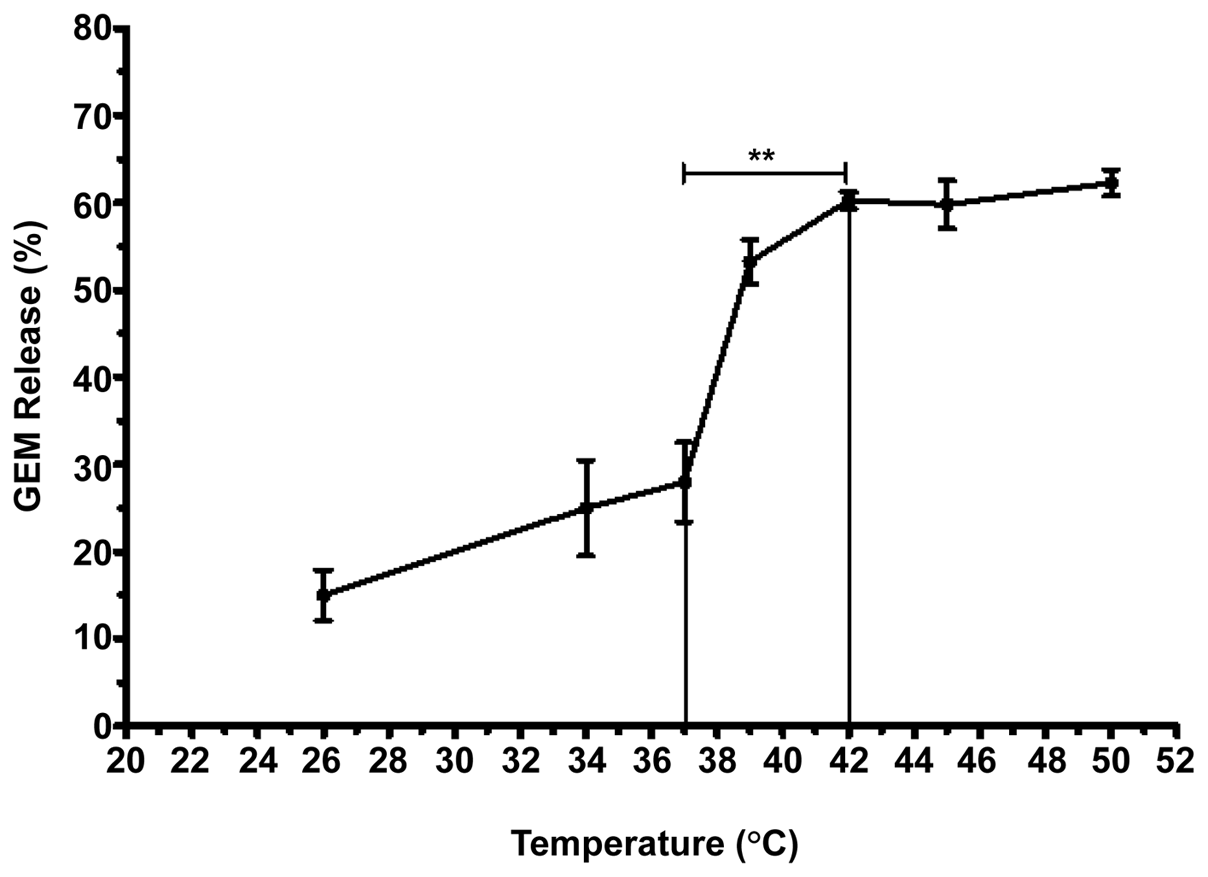
Temperature-dependent release of GEM from TSLnps against different temperatures Different samples were exposed to heat for 10mins and release study was repeated 3 times and data expressed as mean ± S.D, n=3 (**p< 0.01).

Percent release of GEM from TSLnps was determined at different temperatures. Figure 1 shows GEM release pattern as a function of temperature increase. In general, we observed increase in GEM release (%) with increasing temperature. However, a sharped increase in GEM release was noticed between 38°C and 42°C where GEM-TSLnps released about 30% of its content. At 42°C, approximately 60% of GEM was released which is statistically significant (**p<0.01) compared with 25% released at 37°C. Release of GEM was fairly constant after 42°C through to 52°C. The release behavior GEM by TSLnps was consistent with studies conducted by Lim and his colleagues [25].

### Effects of mHT, GEM and GEM-TSLnps + mHT on pancreatic cancer cell lines

#### *In vitro* cell viability studies

**Figure 2:**
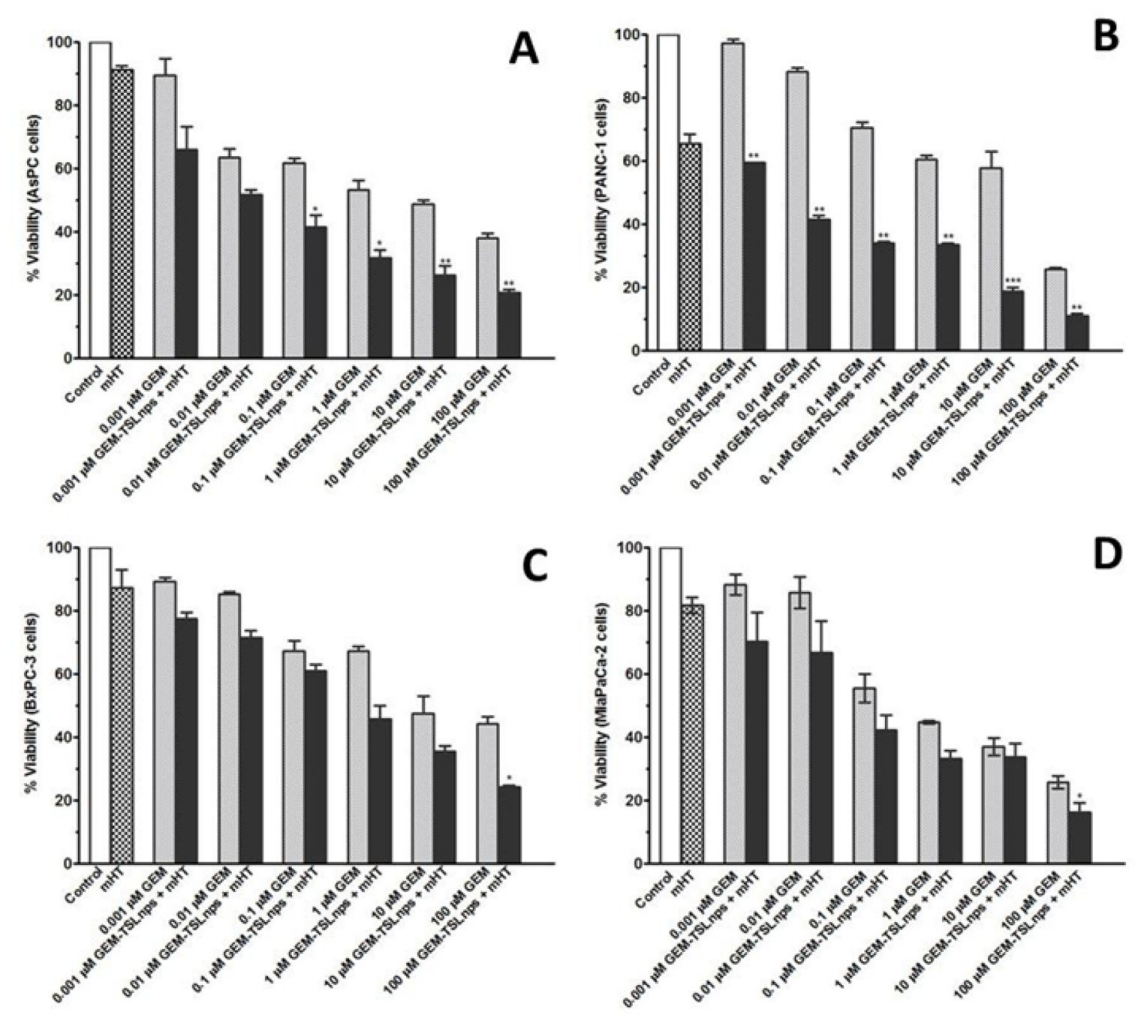
Relationship of % viability between mHT and concentration dependent of GEM from GEM-TSLnps + mHT and GEM cytoxicity in AsPC (A), PANC-1 (B), BxPC-3 (C), and MiaPaCa-2 (D) pancreatic cancer cells. Samples treated with no drug were set to 100% (controls). Results are representative of at least three independent experiments and data expressed as mean ± standard deviation (SD), n=3.

**Figure 3:**
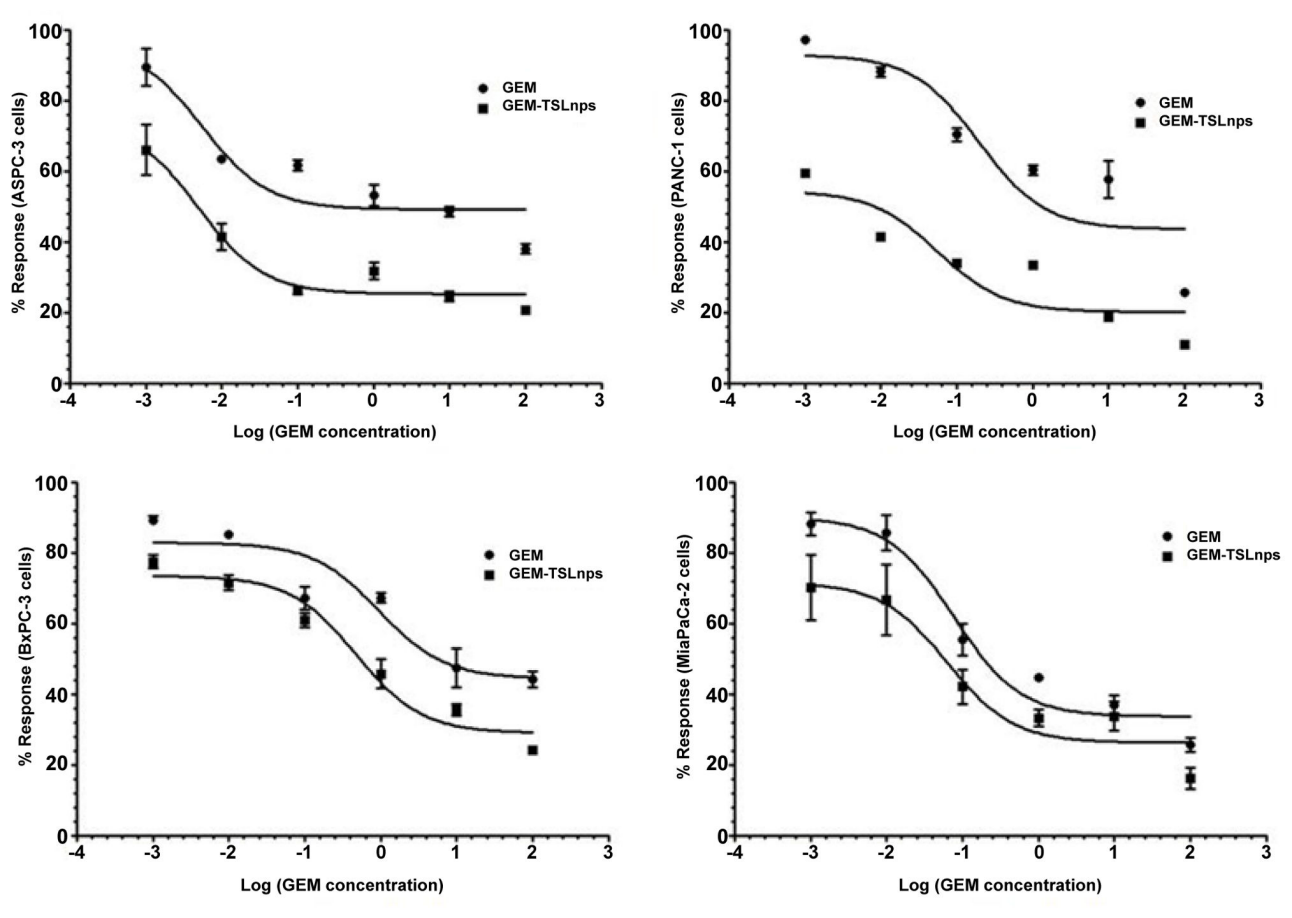
Short-term responses of pancreatic cancer cell lines to GEM and GEM-TSLnps + mHT. Pancreatic cancer cell lines were treated in triplicate with increasing concentrations of GEM and GEM-TSLnps + mHT (0.001 to 100 μM). Non-linear curve fitting for dose-response curves allowed for calculation of an IC_50_ for each (Table 2).

Effects of GEM and GEM-TSLnps + mHT on MiaPaca-2, AsPC, BxPC-3 and PANC-1 cell lines were determined at different concentrations (0.001, 0.01, 0.1, 1, 10 and 100 μM). In general, GEM-TSLnps + mHT treated cell lines showed greater cell growth inhibition than GEM-TSLnps treated cells (Figure 2). No significant effect of mHT alone (exposure time of 10 min at 42°C) was observed on the viability of all the cell lines except PANC-1 cells. Among the GEM-TSLnps + mHT treated cell lines, PANC-1 cells were most sensitive while BxPC-3 cells were less sensitive. Considering GEM treated cell lines, MiaPaCa-2 cells appeared to be most sensitive ones while BxPC-3 cell growth appeared to be less affected by GEM. To determine the effectiveness of GEM-TSLnps + mHT, we correlated its efficacy to its half-maximum inhibitory concentrations (IC_50_) in the pancreatic cancer cell lines (Figure 3) (Table 2). Among the IC_50_ values of GEM-TSLnps + mHT and GEM in the pancreatic cancer cell lines, IC_50_ values in BxPC-3 cells were highest in both treated groups. The lowest IC_50_ value of GEM-TSLnps + mHT was found in AsPC (0.0049 μM) while that of GEM observed in AsPC was 0.0055 μM. No significant difference was observed between IC_50_ values of GEM and GEM-TSLnps + mHT treated AsPC cells. However, IC_50_ values of GEM treated MiaPaCa-2, BxPC-3 and PANC-1cellswere 1.2 to 3.5 fold-higher than that of GEM-TSLnps + mHT treated.

#### Clonogenic survival assay

**Figure 4:**
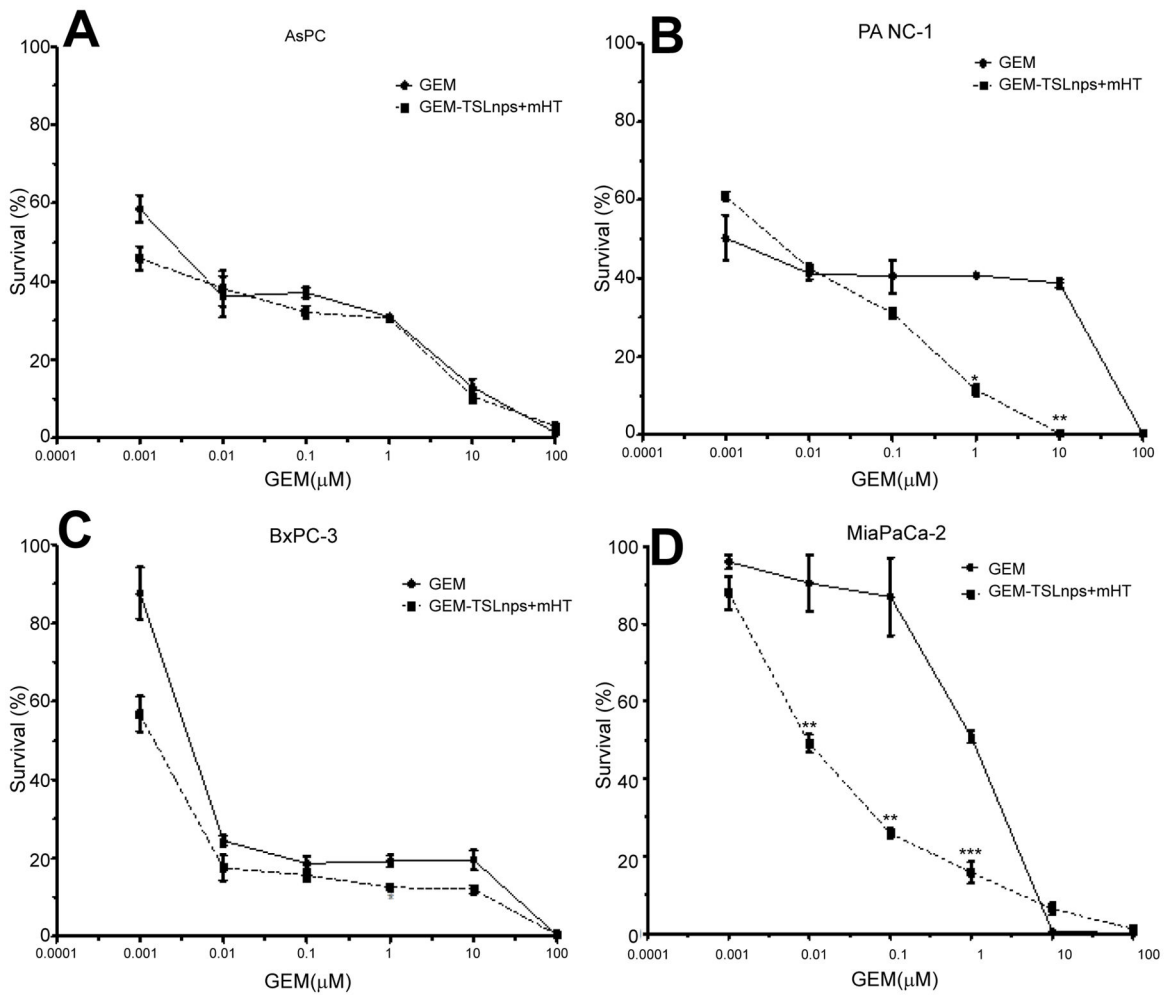
Survival curves of AsPC (A), PANC-1 (B), BxPC-3 (C), and MiaPaCa-2 (D) cells post treatment with GEM (filled circles) and GEM-TSLnp + mHT (filled squares). Data represent mean ± standard deviation (SD), n=3. (*p<0.05, **p<0.01, ***p<0.001).

**Figure 5:**
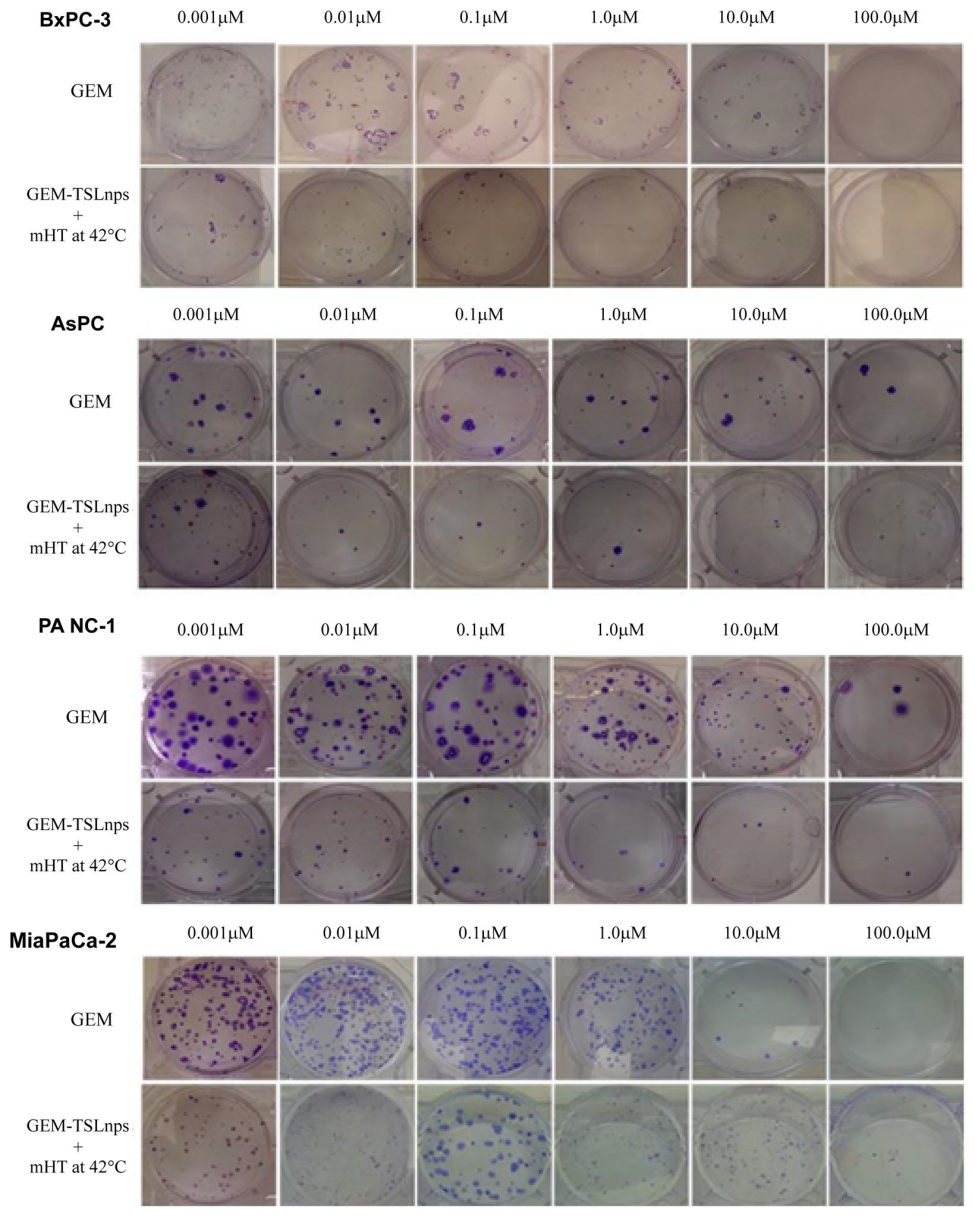
Images of colonies after clonogenic assay on BxPC-3, AsPC, PANC-1 and MiaPaCa-2 cells post-treatment with GEM and GEM-TSLnps + mHT.

The ability of each cell line to retain its proliferative ability and propagate post treatment was tested by the method of clonogenic assay. Dose-dependent survival curves for pancreatic cancer cell lines treated with GEM or GEM-TSLnps + mHT generally showed decreasing cell survival with increase GEM concentration. All GEM-TSLnps + mHT treated cell lines showed a greater reduction in number of colonies formed or % cell survival at a concentration equal to or greater than 0.01 μM compared with GEM (Figure 4). No significant difference between% cell survivals of GEM treated AsPC cells and that of GEM-TSLnps + mHT treated AsPC cells. As expected% cell survival of GEM-TSLnps + mHT treated PANC-1 cells was significantly lower than GEM treated PANC-1 cells with increasing concentration of GEM (p*<0.05 at GEM of 1 μM, p** <0.01 at GEM of 10 μM (Figures 4B, and 5). Although there was no significant difference between% cell viability of GEM μM and GEMTSLnps +mHT in MiaPaCa-2 cells except at GEM concentration at 100 (Figure 2D), we observed a striking difference between of % cell survival of GEM-TSLnps + mHT treated MiaPaCa-2 cells and that of GEM (p**<0.01 for GEM at 0.01 μM and 0.1 μM; p***<0.001 for GEM at 1 μM; Figures 4 and 5).

### Cellular uptake studies by flow cytometry and confocal imaging

**Figure 6:**
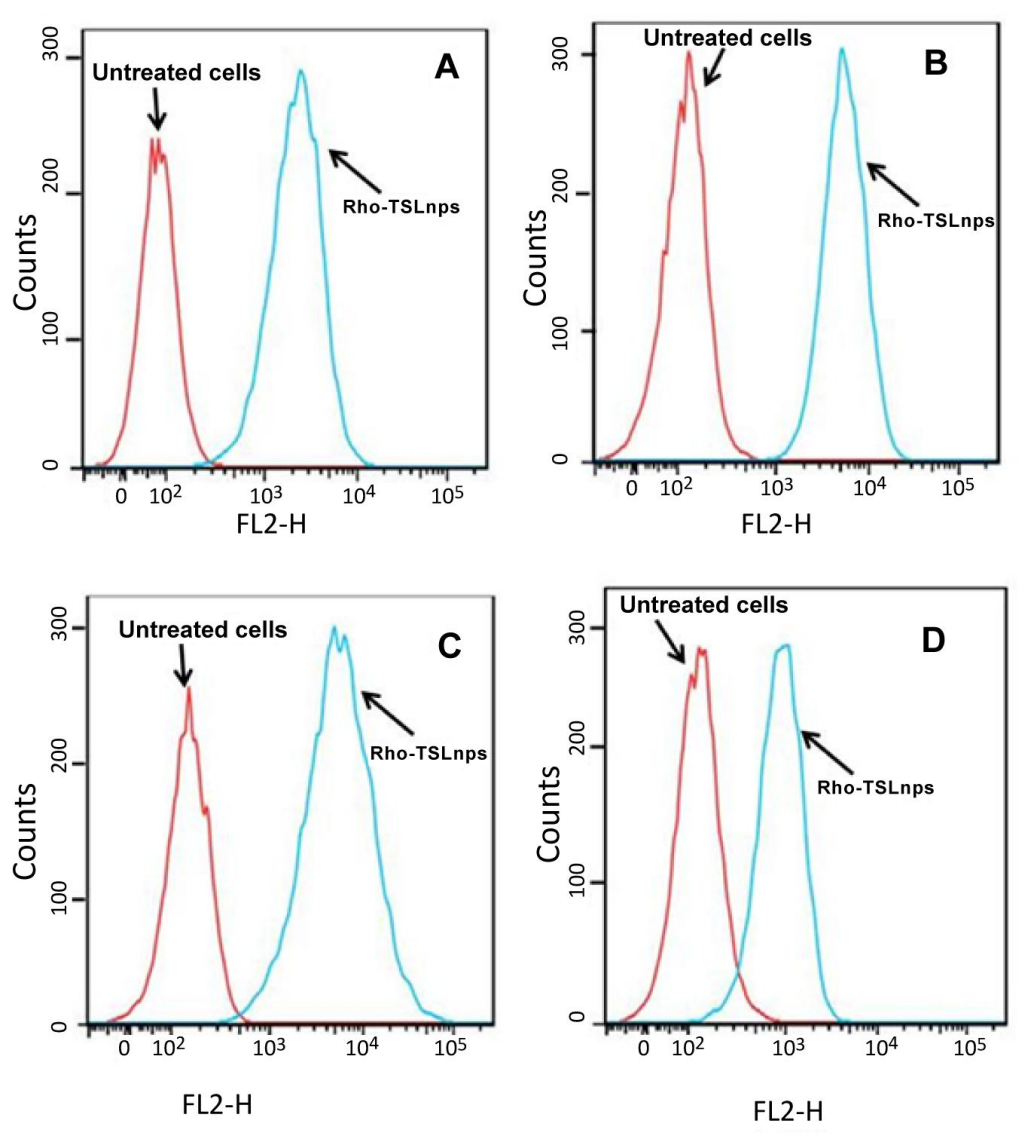
Cellular uptake of Rho-TSLnps by AsPC (A), MiaPaCa-2 (B), PANC-1 (C), and BxPC-3 (D) pancreatic cancer cell lines by flow cytometric analysis.

**Figure 7:**
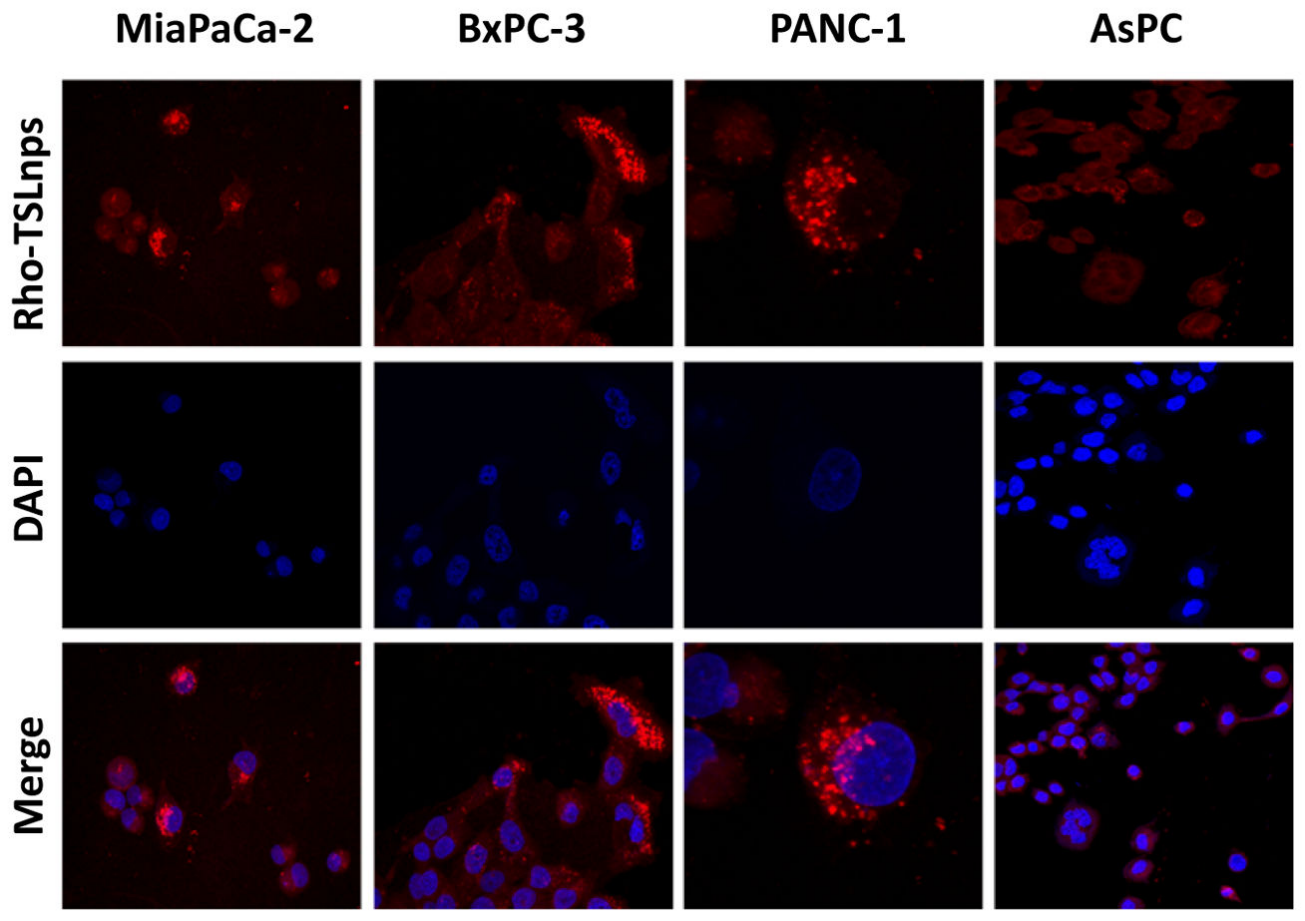
Confocal images showing localization of Rho-TSLnps in pancreatic cancer cell lines after incubation for 4 hr. The nuclei were visualized by staining with DAPI (blue).

Flow cytometric analysis was used to assess the total TSLnps uptake by the AsPC, MiaPaCa-2, PANC-1 and BxPC-3 pancreatic cancer cell lines. Figures 6A, B, C and D show cellular uptake of Rho-TSLnps after cells were incubated for 2 hr at 37°C. A close examination of the flow cytometry data revealed that cellular uptake of Rho-TSLnps by PANC-1 was 1.3-fold higher compared with MiaPaCa-2 or AsPC cells while uptake of Rho-TSLnps by BxPC-3 cells was found to be 0.67-fold lower compared with MiaPaCa-2 or AsPC cells. Despite the differences in the cellular uptake of Rho-TSLnps by these cells, no significant difference was observed among them. To further confirm the uptake of TSLnps by the pancreatic cancer cells, Rho-TSLnps were incubated with AsPC, MiaPaCa-2, PANC-1 and BxPC-3 cells for 24 hr at 37°C and the internalized Rho-TSLnps was imaged using laser confocal microscope. Our data showed a comparable internalization of Rho-TSLnps by all 4 cell lines (Figure 7). Also merged image of Rho-TSLnps and DAPI showed a greater number of Rho-TSLnps localized in the nuclei of the cells.

## Discussion

GEM is the most widely used anticancer drug for treatment of pancreatic cancer. But poor cell membrane permeability and short half-life of 8–17 min have led to repeated administration of the drug to maintain an effective concentration level which is just sufficient to provide palliative treatment or marginally improved survival (20%) [26]. To improve its effectiveness TSLnps were used as carriers to deliver GEM to pancreatic cancer cells.

In this current study, we evaluated the impact of TSLnps on the cytotoxicity enhancement of GEM in vitro by comparing the effects of GEM alone and GEM-TSLnps + mHT in MiaPaCa-2, PANC-1, AsPC and BxPC-3 cell lines. We formulated five different GEM thermosensitive liposomal nanoparticles from which GEM-TSLnp was chosen as the desired nanocarrier based on its ability to entrap high amount of GEM (entrapment efficiency of GEM is 41.10 ± 2.02 (%). Heat triggered release of liposomes was reported to be influenced by lipid composition and melting phase transition temperature (T_m_) [27,28]. At T_m_, the structure of the lipid bilayer changes from a solid gel phase to a liquid-crystalline phase making the membrane more permeable to water and hydrophilic content of liposomes [29]. With DPPC as a major component (86% of lipids total weight, DPPC T_m_ is at 42°C), our TSLnps was found to be significantly stable at 37°C but very unstable around its T_m_ value. This observed behavior of the TSLnps clearly suggests how sensitive they are to temperatures between 39–42°C with sharp release about 60% within 10 min at 42°C. This data is consistent with other temperature sensitive GEM-loaded liposomes [25]. Our in vitro cytotoxic data provided clear evidence to support GEM-TSLnps+ mHT as more effective in pancreatic cancer cell growth inhibition compared with GEM alone.

Out of the 4 pancreatic cancer cell lines that were studied, PANC-1 cells were most sensitive to GEM-TSLnps+ mHT compared with the GEM treated group while BxPC-3 cells were least sensitivity to GEM-TSLnps + mHT. Except PANC-1 cells, we did not observe any appreciable effect of mHT on AsPC, BxPC-3 and MiaPaCa-2 cells compared with the controls. This suggests that mHT barely play any active role in growth inhibition of the three GEM-TSLnps + mHT treated cells but served the purpose of TSLnps disruption and release of its content after internalization in the pancreatic cancer cells. Of course, we partially attributed the significant of PANC-1 cell growth inhibition by GEM-TSLnps + mHT to mHT alone because of its ability to damage about 38% of PANC-1 cells compared with control. We did not observed any significant cell damage to AsPC, BxPC-3 and MiaPaCa-2 cells by mHT, though previous literature reports suggest that heating pancreatic cancer cell lines or other cell lines at 42°C for 60 min can cause irreversible cellular damage [30–32]. We believe that our 10 min mHT treatment of the cell lines was not sufficient to cause any damage to these cells. Further, 10 min was the optimum time required for the TSLnps to release maximum amount of GEM (Figure 1).

For evaluation of short and long-term cytotoxic potential of GEM and GEM-TSLnps + mHT treatment modalities, we conducted viability studies (% viability, Figure 2) for short term and clonogenic assay (% survival, Figure 4) for long-term cytotoxicity. We observed general decrease in cell survival of GEM and GEM-TSLnps+ mHT treated pancreatic cancer cell lines when compared with that of % viability. However, % cell survivals for GEM-TSLnps+ mHT treated PANC-1 and MiaPaCa-2 cells were significantly reduced compared with GEM. These data provide information to assess the differences in the reproductive viability between controls untreated cells and the pancreatic cancer cells that have undergone both GEM and GEMTSLnps+ mHT treatments. Further, the data may be used to determine the effects of cytotoxic agents on colony forming ability in cancer cell lines [33,34].

On the confocal imaging and flow cytometry studies, our findings indicate high uptake of TSLnps by pancreatic cancer cell lines suggesting that TSLnps may interact effectively with plasma membranes of pancreatic cancer cells and deliver its cargo into the cells.

In conclusion, the study provides strong evidence to support TSLnps as an effective drug delivery system that is capable of delivering high amount of GEM. Further, GEM-TSLnps + mHT demonstrated an enhanced anticancer activity in pancreatic cancer cell lines compared with free GEM alone. Additional studies are needed to investigate TSLnps as drug delivery system for GEM and other chemotherapeutic agents in animal studies to determine how effective it can control tumor growth *in vivo*.

## Figures and Tables

**Table 1:** Characterization of gemcitabine-loaded liposomal nanoparticles.

Batch Number	Lipid composition	Molar Ratio	Mean Particle size (nm)	Zeta potential (mV)	Entrapment Efficiency (%)

GEM-TSLnp	DPPC: MPPC: DSPC-PEG_2000_	90:10:4	216.10 ± 0.565	−0.047 ± 0.117	41.10 ± 2.022
GEM-TSLnp_1_	DPPC: DSPC: DSPC-PEG_2000_	26:4:6	204.80 ± 0.499	0.623 ± 0.110	9.48 ± 0.475
GEM-TSLnp_2_	HSPC: CHOL: DSPC-PEG_2000_	75:50:3	285.00 ± 0.442	0.018 ± 0.678	7.73 ± 0.258
GEM-TSLnp_3_	DPPC: DSPC: DSPC-PEG_2000_	80:15:5	159.70 ± 0.388	0.153 ± 0.025	3.01 ± 1.277
GEM-TSLnp_4_	DPPC: HSPC:CHOL:DSPC-PEG_2000_	100:50:30:6	155.80 ± 0.320	1.090 ± 0.023	2.94 ± 0.464

**Table 2:** IC_50_ values (μM) of cytotoxic GEM and GEM-TSLnps in pancreatic cancer cell lines.

Cell lines	GEM		GEM-TSLnps + mHT	

	IC_50_	(95% CI)	IC_50_	(95% CI)
MiaPaCa-2	0.077	(0.0302–0.1948)	0.063	(0.0116–0.3458)
PANC-1	0.195	(0.0266–1.422)	0.056	(0.007–0.4085)
BxPC-3	0.943	(0.2066–4.308)	0.475	(0.1825–1.233)
AsPC	0.0055	(0.0009–0.0327)	0.0049	(0.0014–0.0167)

## References

[1] Siegel RL, Miller KD, Jemal A (2015). Cancer statistics, 2015. CA Cancer J Clin.

[2] Cancer Facts and Figures 2015.

[3] Mahalingam D, Giles F (2008). Challenges in developing targeted therapy for pancreatic adenocarcinoma. Expert Opin Ther Targets.

[4] Iacobuzio-Donahue CA, Fu B, Yachida S, Luo M, Abe H (2009). DPC4 gene status of the primary carcinoma correlates with patterns of failure in patients with pancreatic cancer. J Clin Oncol.

[5] Wu NZ, Da D, Rudoll TL, Needham D, Whorton AR (1993). Increased microvascular permeability contributes to preferential accumulation of Stealth liposomes in tumor tissue. Cancer Res.

[6] Wong C, Stylianopoulos T, Cui J, Martin J, Chauhan VP (2011). Multistage nanoparticle delivery system for deep penetration into tumor tissue. Proc Natl Acad Sci U S A.

[7] Moore MJ, Goldstein D, Hamm J, Figer A, Hecht JR (2007). Erlotinib plus gemcitabine compared with gemcitabine alone in patients with advanced pancreatic cancer: a phase III trial of the National Cancer Institute of Canada Clinical Trials Group. J Clin Oncol.

[8] Immordino ML, Brusa P, Rocco F, Arpicco S, Ceruti M (2004). Preparation, characterization, cytotoxicity and pharmacokinetics of liposomes containing lipophilic gemcitabine prodrugs. J Control Release.

[9] Lameire N (2014). Nephrotoxicity of recent anti-cancer agents. Clin Kidney J.

[10] Bender DM, Bao J, Dantzig AH, Diseroad WD, Law KL (2009). Synthesis, crystallization, and biological evaluation of an orally active prodrug of gemcitabine. J Med Chem.

[11] Koay EJ, Truty MJ, Cristini V, Thomas RM, Chen R (2014). Transport properties of pancreatic cancer describe gemcitabine delivery and response. J Clin Invest.

[12] Jain RK (1998). Delivery of molecular and cellular medicine to solid tumors. J Control Release.

[13] Jain RK (2008). Taming vessels to treat cancer. Sci Am.

[14] Jain RK, Stylianopoulos T (2010). Delivering nanomedicine to solid tumors. Nat Rev Clin Oncol.

[15] Boucher Y, Baxter LT, Jain RK (1990). Interstitial pressure gradients in tissue-isolated and subcutaneous tumors: implications for therapy. Cancer Res.

[16] Ta T, Porter TM (2013). Thermosensitive liposomes for localized delivery and triggered release of chemotherapy. J Control Release.

[17] Achim M, Precup C, Gonganaunitu D, Barbu-Tudoran L, Porfire Alina Silvia (2009). Thermosensitive liposomes containing doxorubicin. preparation and in vitro evaluation. Farmacia.

[18] Li L, ten Hagen TL, Hossann M, Suss R, van Rhoon GC (2013). Mild hyperthermia triggered doxorubicin release from optimized stealth thermosensitive liposomes improves intratumoral drug delivery and efficacy. J Controlled Release.

[19] Needham D, Anyarambhatla G, Kong G, Dewhirst MW (2000). A new temperature-sensitive liposome for use with mild hyperthermia: characterization and testing in a human tumor xenograft model. Cancer Res.

[20] Lanz C, Früh M, Thormann W, Cerny T, Lauterburg BH (2007). Rapid determination of gemcitabine in plasma and serum using reversed-phase HPLC. J Sep Sci.

[21] Celano M, Calvagno MG, Bulotta S, Paolino D, Arturi F (2004). Cytotoxic effects of gemcitabine-loaded liposomes in human anaplastic thyroid carcinoma cells. BMC Cancer.

[22] Franken NA, Rodermond HM, Stap J, Haveman J, van Bree C (2006). Clonogenic assay of cells in vitro. Nat Protoc.

[23] Molejon MI, Tellechea JI, Loncle C, Gayet O, Gilabert M (2015). Deciphering the cellular source of tumor relapse identifies CD44 as a major therapeutic target in pancreatic adenocarcinoma. Oncotarget.

[24] Huth US, Schubert R, Peschka-Süss R (2006). Investigating the uptake and intracellular fate of pH-sensitive liposomes by flow cytometry and spectral bio-imaging. J Control Release.

[25] Lim SK, Shin DH, Choi MH, Kim JS (2014). Enhanced antitumor efficacy of gemcitabine-loaded temperature-sensitive liposome by hyperthermia in tumor-bearing mice. Drug Dev Ind Pharm.

[26] Schoppmeyer K, Kronberg J, Tannapfel A, Mossner J, Wittekind C (2005). Predictive value of heparanase expression in the palliative therapy of pancreatic cancer. Pancreatology.

[27] Hossann M, Wiggenhorn M, Schwerdt A, Wachholz K, Teichert N (2007). In vitro stability and content release properties of phosphatidylglyceroglycerol containing thermosensitive liposomes. Biochim Biophys Acta.

[28] Kneidl B, Peller M, Winter G, Lindner LH, Hossann M (2014). Thermosensitive liposomal drug delivery systems: state of the art review. Int J Nanomedicine.

[29] Grull H, Langereis S (2012). Hyperthermia-triggered drug delivery from temperature-sensitive liposomes using MRI-guided high intensity focused ultrasound. J Control Release.

[30] Milanovic D, Firat E, Grosu AL, Niedermann G (2013). Increased radiosensitivity and radiothermosensitivity of human pancreatic MIA PaCa-2 and U251 glioblastoma cell lines treated with the novel Hsp90 inhibitor NVP-HSP990. Radiat Oncol.

[31] Guo Y, Ziesch A, Hocke S, Kampmann E, Ochs S (2015). Overexpression of heat shock protein 27 (HSP27) increases gemcitabine sensitivity in pancreatic cancer cells through S-phase arrest and apoptosis. J Cell Mol Med.

[32] Adachi S, Kokura S, Okayama T, Ishikawa T, Takagi T (2009). Effect of hyperthermia combined with gemcitabine on apoptotic cell death in cultured human pancreatic cancer cell lines. Int J hyperthermia.

[33] Sumantran VN (2011). Cellular chemosensitivity assays: an overview. Methods Mol Biol.

[34] Florento L, Matias R, Tuano E, Santiago K, Dela Cruz F (2012). Comparison of Cytotoxic Activity of Anticancer Drugs against Various Human Tumor Cell Lines Using In Vitro Cell-Based Approach. Int J Biomed Sci.

